# Familial co-aggregation and shared genetics of cardiometabolic disorders and traits: data from the multi-generational Lifelines Cohort Study

**DOI:** 10.1186/s12933-023-02017-w

**Published:** 2023-10-21

**Authors:** Rima D. Triatin, Zekai Chen, Alireza Ani, Rujia Wang, Catharina A. Hartman, Ilja M. Nolte, Chris H. L. Thio, Harold Snieder

**Affiliations:** 1 Department of Epidemiology, University Medical Center Groningen, University of Groningen, Hanzeplein 1, P.O. Box 30.001 (FA40), 9700RB Groningen, The Netherlands; 2 Department of Biomedical Sciences, Faculty of Medicine, Universitas Padjadjaran, Bandung, Indonesia; 3 Department of Bioinformatics, Isfahan University of Medical Sciences, Isfahan, 8174673461 Iran; 4 Department of Psychiatry, Interdisciplinary Center Psychopathology and Emotion Regulation (ICPE), University Medical Center Groningen, University of Groningen, Groningen, The Netherlands; 5 Department of Population Health Sciences, Institute for Risk Assessment Sciences (IRAS), Utrecht University, Utrecht, The Netherlands

**Keywords:** Cardiometabolic disorders, Cardiometabolic traits, Familial (co-)aggregation, Genetic correlation, Heritability

## Abstract

**Background:**

It is unclear to what extent genetics explain the familial clustering and the co-occurrence of distinct cardiometabolic disorders in the general population. We therefore aimed to quantify the familial (co-)aggregation of various cardiometabolic disorders and to estimate the heritability of cardiometabolic traits and their genetic correlations using the large, multi-generational Lifelines Cohort Study.

**Methods:**

We used baseline data of 162,416 participants from Lifelines. Cardiometabolic disorders including type 2 diabetes (T2D), cardiovascular diseases, hypertension, obesity, hypercholesterolemia, and metabolic syndrome (MetS), were defined in adult participants. Fifteen additional cardiometabolic traits indexing obesity, blood pressure, inflammation, glucose regulation, and lipid levels were measured in all included participants. Recurrence risk ratios (λ_R_) for first-degree relatives (FDR) indexed familial (co-)aggregation of cardiometabolic disorders using modified conditional Cox proportional hazards models and were compared to those of spouses. Heritability (h^2^), shared environment, and genetic correlation (r_g_) were estimated using restricted maximum likelihood variance decomposition methods, adjusted for age, age^2^, and sex.

**Results:**

Individuals with a first-degree relative with a cardiometabolic disorder had a higher risk of the same disorder, ranging from λ_FDR_ of 1.23 (95% CI 1.20–1.25) for hypertension to λ_FDR_ of 2.48 (95% CI 2.15–2.86) for T2D. Most of these were higher than in spouses (λ_Spouses_ < λ_FDR_), except for obesity which was slightly higher in spouses. We found moderate heritability for cardiometabolic traits (from h^2^_CRP_: 0.26 to h^2^_HDL_: 0.50). Cardiometabolic disorders showed positive familial co-aggregation, particularly between T2D, MetS, and obesity (from λ_FDR obesity-MetS_: 1.28 (95% CI 1.24–1.32) to λ_FDR MetS-T2D_: 1.61 (95% CI 1.52–1.70)), consistent with the genetic correlations between continuous intermediate traits (ranging from r_g HDL-Triglycerides_: − 0.53 to r_g LDL-Apolipoprotein B_: 0.94).

**Conclusions:**

There is positive familial (co-)aggregation of cardiometabolic disorder, moderate heritability of intermediate traits, and moderate genetic correlations between traits. These results indicate that shared genetics and common genetic architecture contribute to cardiometabolic disease.

**Supplementary Information:**

The online version contains supplementary material available at 10.1186/s12933-023-02017-w.

## Background

Cardiometabolic disorders, such as type 2 diabetes (T2D), cardiovascular diseases (CVD), obesity, hypercholesterolemia, and hypertension are interrelated conditions, responsible for high mortality and disability rates worldwide. The global burden of these disorders is continuously increasing: the global population with CVD doubled from 271 million in 1990 to 523 million in 2019 [1], and the case-number of diabetes (20–79 years) is predicted to increase from 537 million in 2021 to 783 million in 2045 [2]. These cardiometabolic disorders often co-occur within individuals, suggesting a co-pathogenesis of metabolic abnormalities among various cardiometabolic disorders, which is commonly described as metabolic syndrome (MetS), a highly prevalent, multifaceted cluster of metabolic abnormalities [3–9].

A role of genetics is likely: several studies have observed familial aggregation of CVD [10, 11] and T2D [12], obesity [13, 14], and MetS [15–17], while cardiometabolic traits, such as blood pressure, fasting blood glucose, and total cholesterol, were shown to be heritable [18–20]. Some evidence exists that different cardiometabolic disorders co-aggregate within families, i.e., a family history of a specific cardiometabolic disorder associates with elevated risk of another cardiometabolic disorder [21–24]. However, familial aggregation, and especially co-aggregation of a full spectrum of cardiometabolic disorders, has not yet been investigated comprehensively within a single study. Furthermore, the accuracy and generalizability of most family studies are limited by various factors, such as modest sample size ranging from 2302 to 17,954 individuals, specific founder populations, different family relationship included (e.g., only siblings or parent-offspring), or the use of self-reported family history not validated by objective measures.

For above reasons, it remains uncertain to what extent cardiometabolic disorders (co-)aggregate in families in the general population, and to what extent the correlation between cardiometabolic disorders and traits can be explained by genetics. Bridging this knowledge gap may help risk stratification and early detection of cardiometabolic disorders. Furthermore, knowledge of shared genetics between disorders and traits may help advance our understanding of pathophysiology. Therefore, we aimed to: (1) quantify the familial (co-)aggregation of various cardiometabolic disorders; (2) estimate the heritability of a wide array of underlying cardiometabolic traits; and (3) estimate genetic correlations between cardiometabolic traits, by using extensive data from Lifelines, a large multi-generational family study representative of the general Dutch population.

## Methods

### Study population

Lifelines is a multi-disciplinary prospective population-based cohort study examining in a unique three-generation design the health and health-related behaviours of 167,729 persons living in the North of the Netherlands. It employs a broad range of investigative procedures in assessing the biomedical, socio-demographic, behavioural, physical and psychological factors which contribute to the health and disease of the general population, with a special focus on multi-morbidity and complex genetics [25]. The recruitment of participants and their families, and how we define household ID are detailed in the Supplementary Methods (Additional file 1). Briefly, kinship was registered by questionnaires and verified where possible in participants with genetic data (n ~ 80,000). Participants who lived in the same house, which was determined based on registered postal codes, shared the same household ID. In total, there were 30,914 families (of size ≥ 2) of up to four generations and 40,496 singletons (i.e., participants without any relative participating in Lifelines). Physical measurements and collection of biological samples were performed in participants aged 8 years and older [25].

A total of 162,416 participants aged 8 to 93 (152,723 adults and 9693 children) were included in the current cross-sectional study. Data on six cardiometabolic disorders and fifteen cardiometabolic traits were extracted from the database. Cardiometabolic disorders included T2D, MetS, hypertension, hypercholesterolemia, obesity, and CVD. Cardiometabolic traits included markers of glucose regulation (fasting blood glucose, glycated haemoglobin [HbA1c] and skin autofluorescence), blood pressure (systolic and diastolic blood pressure [SBP and DBP]), inflammation (leukocyte count and c-reactive protein [CRP]), obesity (body mass index [BMI] and waist circumference), and lipid levels (total cholesterol, high density lipoprotein cholesterol [HDL], low density lipoprotein cholesterol [LDL], apolipoprotein A, apolipoprotein B, and triglycerides).

Signed informed consent was provided by all participants. Lifelines was conducted according to the principles of the Declaration of Helsinki and following the research code of the University Medical Center Groningen. Meanwhile, the study was approved by the medical ethical committee of the University Medical Center Groningen.

### Measurement

#### Cardiometabolic disorders

Cardiometabolic disorders were defined in adult participants and included T2D, MetS, hypertension, hypercholesterolemia, obesity, and CVD. Individuals with at least one of these six conditions were considered prevalent cases of any cardiometabolic disorder.

##### Type 2 diabetes

We defined T2D as the combination of self-reported T2D and any supporting data (i.e., use of self-reported glucose-lowering medication, fasting plasma glucose level ≥ 7.0 mmol/L, and/or HbA_1c_ ≥ 6.5%). The following participants were not considered to have T2D: (1) self-reported diabetes but without support by laboratory and medication data; (2) self-reported diabetes but age < 30 years at the time of visit; (3) self-reported diabetes but reported age of onset < 30 years old with self-reported insulin medication.

##### Metabolic syndrome

According to National cholesterol Education Program Adult Treatment Panel III (NCEP ATP III) definition, participants with three or more of the five following criteria were defined as having MetS: (1) SBP ≥ 130 mmHg, and/or DBP ≥ 85 mmHg, and/or use of antihypertensive medication (Anatomical Therapeutic Chemical [ATC] Classification Codes C02, C03, C07, C08, and/or C09; a key to the ATC codes can be found in the Additional file 2: Table S9); (2) fasting blood glucose ≥ 5.6 mmol/L, and/or use of blood glucose-lowering medication, and/or diagnosis of T2D; (3) HDL cholesterol levels < 1.03 mmol/L in men, and < 1.30 mmol/L in women, and/or use of lipid-lowering medication (ATC codes C10A and/or C10B); (4) triglyceride levels ≥ 1.70 mmol/L and/or use of lipid-lowering medication (ATC codes C10A and/or C10B); and (5) waist circumference ≥ 102 cm in men and ≥ 88 cm in women [26].

##### Hypertension

Hypertension was defined by SBP ≥ 140 mmHg, and/or DBP ≥ 90 mmHg, and/or the use of antihypertensive medication (ATC codes C02, C03, C07, C08, C09, and/or G04CA03).

##### Hypercholesterolemia

Total cholesterol ≥ 6.5 mmol/L and/or the use of lipid-lowering medication (ATC codes C10A and/or C10B) were used to define hypercholesterolemia. For participants with self-reported myocardial infarction, total cholesterol ≥ 5.0 mmol/L was used as a cut off to define hypercholesterolemia [27].

##### Obesity

BMI was calculated as weight (kg)/height squared (m^2^). BMI ≥ 30 was used to define obesity in adults [28].

##### Cardiovascular diseases

Four types of CVD were used to define CVD cases at baseline, including myocardial infarction with drugs (platelet aggregation inhibitors/antithrombotic drugs) or ECG abnormalities, self-reported heart failure with drug use (angiotensin converting enzyme inhibitors/angiotensin-II receptor antagonists/aldosterone antagonists) or therapy (pacemaker, ICD implantation or heart transplant), self-reported stroke, and self-reported cardiac surgery (i.e., Coronary Arterial By-pass Graft, Percutaneous Transluminal Coronary Angioplasty, and stent positioning). Participants with at least one of these four CVD were considered prevalent cases. To investigate a broader range of CVDs, we also used an extended CVD definition previously reported by van der Ende et al. [27]. The extended CVD definition additionally included self-reported heart valve problems, self-reported atherosclerosis, self-reported thrombosis, self-reported aneurysm, narrowing carotids, atrial fibrillation with drugs or CHA_2_DS_2_-VASc (congestive heart failure, hypertension, age ≥ 75 (doubled), diabetes mellitus, prior stroke or transient ischemic attack (doubled), vascular disease, age 65–74, female) score > 2, and self-reported arrhythmia.

#### Cardiometabolic traits

Biomarkers were measured from fasting blood samples at the laboratory centre of the University Medical Center Groningen. Skin autofluorescence was measured in adults during baseline visits to quantify the accumulation of Advanced Glycation End products in the skin. Anthropometrics, including body weight, body height, and waist circumference, were performed by a trained research nurse, following the Lifelines protocol [29]. SBP and DBP were measured repetitively 10 times within 10 min, but only the average of the last 3 measurements was used for analysis. Details of the cardiometabolic trait measurements are explained in Additional file 1: Supplementary Methods.

### Statistical analysis

To adjust for treatment effects, 15 mmHg and 10 mmHg were added to the SBP and DBP values, respectively, in individuals taking antihypertensive medication. This method has been shown to reduce bias and improve statistical power [30]. Also, for individuals taking lipid-lowering medication, total cholesterol and LDL-C were adjusted by dividing their value by 0.8 and 0.7, respectively [31]. Data was presented depending on the type of variables and its distribution. Continuous variables were presented as mean ± SD when normally distributed and as median and interquartile range when non-normally distributed. Binary cardiometabolic disorders at baseline were described by the number of cases and their prevalences. All analyses were conducted in R version 4.2.2.

#### Familial aggregation and co-aggregation

Familial aggregation and co-aggregation of cardiometabolic disorders were quantified by the recurrence risk ratio (λ_R_) introduced by Risch [32], and previously applied in Lifelines [13, 33]. The recurrence risk ratio is defined as a ratio between the risk in those with an affected first-degree relative (FDR) and the risk of the total Lifelines population, with λ_R_ > 1 indicating positive familial aggregation (i.e. elevated risk in those with positive family history). This λ_R_ was estimated using a modified, conditional Cox proportional hazards model applied to cross-sectional data. The modification of this model was performed by applying equal time-to-event for all participants [34]. This model was adjusted for age, age^2^ (to account for non-linear age effects), sex, and accounted for within-family correlations. We repeated this analysis on spouses as a negative control, as spouses are unlikely to be genetically related and estimates of λ_R_ therefore represent the general influence of shared environmental factors and/or assortative mating on cardiometabolic disorders.

As exploratory analyses, we performed familial aggregation analyses of the six cardiometabolic disorders stratified by age, sex, and familial relationship.

#### Heritability, genetic correlation and phenotypic correlation

Heritability (h^2^) was defined as the ratio of additive genetic variance to the total phenotypic variance (h^2^ = V_A_/V_P_), using family pedigree as a proxy. The contribution of household (or shared/common environment, c^2^) was defined as c^2^ = V_C_/V_P_. We estimated h^2^ and c^2^ of cardiometabolic traits using residual maximum likelihood-based (REML) variance decomposition under linear mixed models implemented in the ASReml-R package (ASReml, UK) [35]. Significance level of this estimate was derived from likelihood ratio tests, comparing the heritability model to a model in which additive genetic variances was constrained to zero. A detailed description of testing the significance of genetic correlations and phenotypic correlations can be found in the Supplementary Methods (Additional file 1).

Bivariate REML-based variance decomposition was used to estimate the genetic and phenotypic correlation between pairs of cardiometabolic traits. Genetic correlations between traits were estimated as: $r_G=\frac{{\sigma}_{A1A2}}{\sqrt{{\sigma }_{A1}^{2}{\sigma }_{A2}^{2}}}$, where σ_A1A2_ is the estimated additive genetic covariance between trait x and trait y, and σ^2^_A1_ and σ^2^_A2_ are the estimated additive genetic variances for traits x and y, respectively. Phenotypic correlations between pairs of traits were obtained from estimated phenotypic covariance and variance as: $r_P=\frac{{\sigma }_{P1P2}}{\sqrt{{\sigma }_{P1}^{2}{\sigma }_{P2}^{2}}}$, where σ_P1P2_ is the phenotypic covariance between trait x and trait y, and σ^2^_P1_ and σ^2^_P2_ is the phenotypic variance for traits x and y, respectively.

A rank-based inverse-normal transformation was applied to all variables prior to analysis. In addition, we performed log-transformation on non-normally distributed variables as a sensitivity analysis to further validate our results. All models for heritability, genetic correlations, and phenotypic correlations were adjusted for age, age^2^ and sex.

## Results

### Basic characteristics

**Fig. 1:**
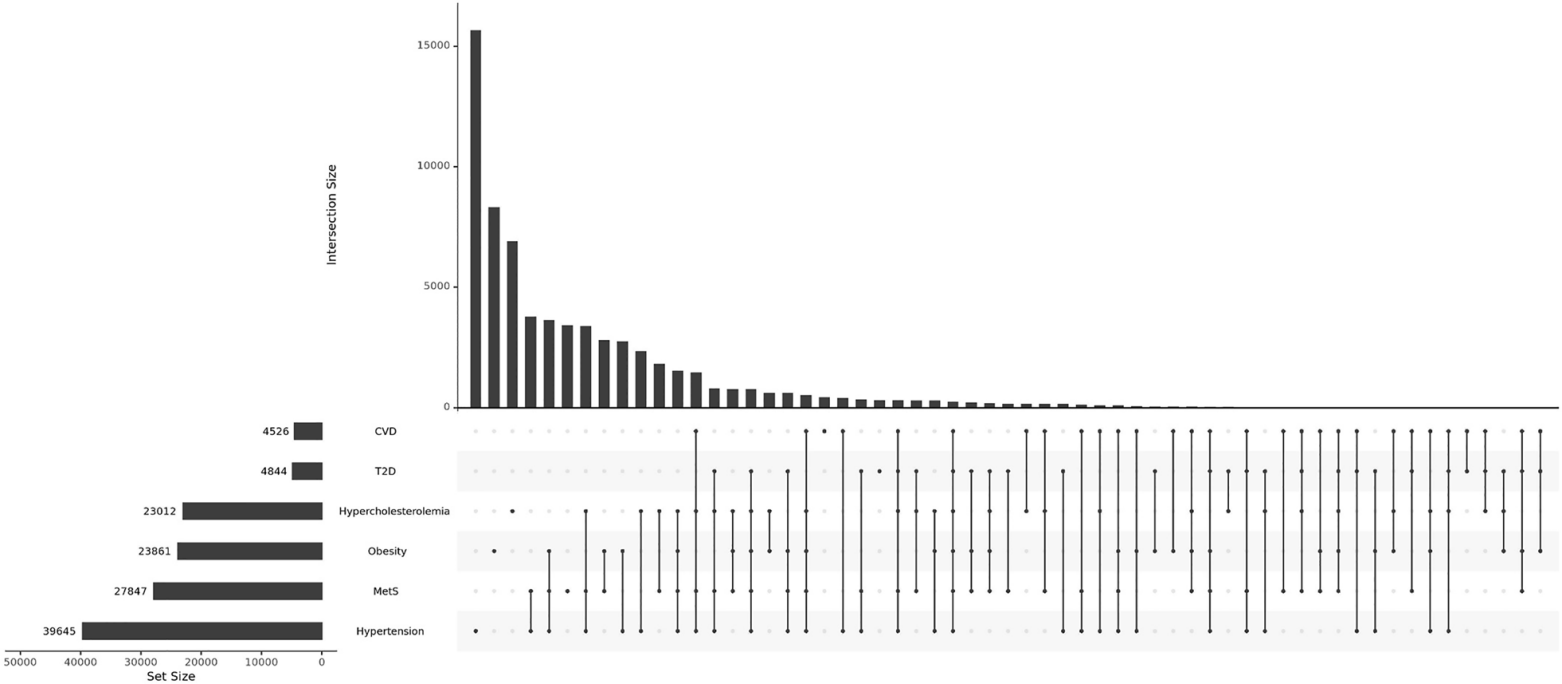
Upset plot showing the overlap in the prevalence of cardiometabolic disorders. The bars in the lower left panel show the total number of cases of each cardiometabolic disorder. The connected black dots in the matrix panel below represent the combination of multiple cardiometabolic disorders in participants, while a single dot without connection to others indicates a single morbidity in participants. The upper bars, representing the intersection size, show the number of individuals with cardiometabolic disorders and its comorbidities highlighted by the connected black dots in the matrix panel below

Of the total of 152,723 included adult participants, 66,695 (43.7%) had at least one cardiometabolic disorder, of which approximately half had multiple (≥ 2) morbidities, shown in Table 1. The prevalence of co-occurring cardiometabolic disorders within individuals is illustrated in Fig. 1. The most common comorbidities in our study population involved a combination of MetS and hypertension with a prevalence of 2.47% among the total participants. Given the definition of MetS, it is possible that MetS overlaps with other cardiometabolic disorders. However, this overlap is incomplete, given that 12.28% of MetS cases only had a single morbidity without any other investigated morbidities (i.e. obesity, hypertension, CVD, T2D, hypercholesterolemia), and less than 1% of MetS cases had all morbidities (details in Additional file 2: Table S1). Meanwhile, 93.4% of participants with T2D had at least one comorbidity, as shown in Additional file 1: Fig. S1). In addition, the prevalence of all types of cardiometabolic disorders was expectedly higher in the older age group (Additional file 1: Fig. S2).

**Table 1:** Characteristics of the Lifelines participants (N = 162,416)

Baseline characteristic	All participants (N = 162,416)			Adults (N = 152,723)			Children (N = 9693)		
	N	n/mean ± SD/median (IQR)	Prevalence (%)	N	n/mean ± SD/median (IQR)	Prevalence (%)	N	n/mean ± SD/median (IQR)	Prevalence (%)
Age (years)	162,416	42.68 ± 14.92		152,723	44.63 ± 13.13		9693	11.97 ± 2.77	
Gender (female)	162,416	94,428	58.1	152,723	89,340	58.5	9693	5088	52.5
Any cardiometabolic disorder				152,723	66,695	43.7	N/A		
Single disease				152,723	35,075	23	N/A		
Multi-morbidities (>1 diseases)				152,723	31,620	20.7	N/A		
*Cardiometabolic disorders*									
One or more cardiovascular diseases				152,230	4526	3	N/A		
MI with drug use or ECG abnormalities				152,230	1881	1.2	N/A		
Self-reported heart failure with drug use or therapy				152,230	802	0.5	N/A		
Self-reported cardiac surgery (PTCA, CABG, and stent positioning)				152,230	2221	1.5	N/A		
Self-reported stroke				152,230	1178	0.8	N/A		
Type 2 diabetes				152,341	4844	3.2	N/A		
Hypertension				151,342	39,645	26.2	N/A		
Obesity				152,588	23,861	15.6	N/A		
Hypercholesterolemia				147,785	23,012	15.6	N/A		
Metabolic syndrome				144,400	27,847	19.3	N/A		
*Cardiometabolic traits*									
Waist circumference (cm)	162,278	88.80 ± 13.48		152,588	90.16 ± 12.51		9690	67.30 ± 9.32	
Body weight (Kg)	162,279	77.86 ± 17.11		152,588	79.77 ± 15.33		9691	47.70 ± 15.23	
Body height (cm)	162,281	173.75 ± 10.72		152,590	174.79 ± 9.43		9691	157.50 ± 15.65	
BMI (Kg/m^2^)	162,279	25.62 ± 4.63		152,588	26.06 ± 4.35		9691	18.72 ± 3.19	
Systolic Blood Pressure* (mmHg)	162,306	126.04 ± 17.53		152,628	127.29 ± 17.13		9678	106.43 ± 10.82	
Diastolic Blood Pressure* (mmHg)	162,306	73.96 ± 10.77		152,628	74.88 ± 10.33		9678	59.51 ± 6.28	
Skin auto fluorescence (z-score)	83,057	1.93 ± 0.44		83,057	1.93 ± 0.44		N/A		
C-reactive protein (mg/l)	49,924	1.2 (0.00–247.00)		49,487	1.2 (0.0–247.0)		437	0.5 (0.20–69.50)	
Leukocyte count (10^9^/l)	155,477	5.8 (1.20–126.60)		147,353	5.8 (1.2–126.6)		8124	5.5 (1.60–17.40)	
Total cholesterol*(mmol/l)	155,855	6.27 ± 1.27		147,658	6.33 ± 1.26		8197	5.10 ± 0.85	
Triglycerides (mmol/l)	155,855	0.96 (0.01–37.31)		147,658	0.98 (0.01–37.31)		8197	0.65 (0.11–8.11)	
HDL cholesterol (mmol/l)	155,854	1.49 ± 0.39		147,657	1.49 ± 0.40		8197	1.55 ± 0.33	
LDL cholesterol* (mmol/l)	155,845	4.43 (0.17–18.86)		147,648	4.57 (0.14–18.86)		8197	3.29 (0.14–10.29)	
Apolipoprotein A (mmol/l)	42,103	1.54 ± 0.27		41,751	1.54 ± 0.27		352	1.40 ± 0.20	
Apolipoprotein B (mmol/l)	42,090	0.92 ± 0.24		41,738	0.93 ± 0.24		352	0.64 ± 0.16	
HbA1c (%)	154,818	5.50 (2.50–16.40)		146,755	5.50 (2.50–16.40)		8063	5.40 (2.80–15.00)	
Fasting blood glucose (mmol/l)	151,530	4.90 (1.90–24.40)		143,640	4.90 (1.90–24.40)		7890	4.60 (2.00–18.30)	

CABG: coronary artery bypass graft; MI: myocardial infarction; N/A: not available; PTCA: percutaneous transluminal coronary angioplasty

^*^Values are adjusted for anti-hypertensive or lipid-lowering medication. For systolic blood pressure 15 mmHg was added and for diastolic blood pressure 10 mmHg was added in individuals taking anti-hypertensive medication, while total cholesterol was divided by 0.8 and LDL cholesterol were divided by 0.7 in individuals taking lipid lowering medication

### Familial aggregation

**Fig. 2:**
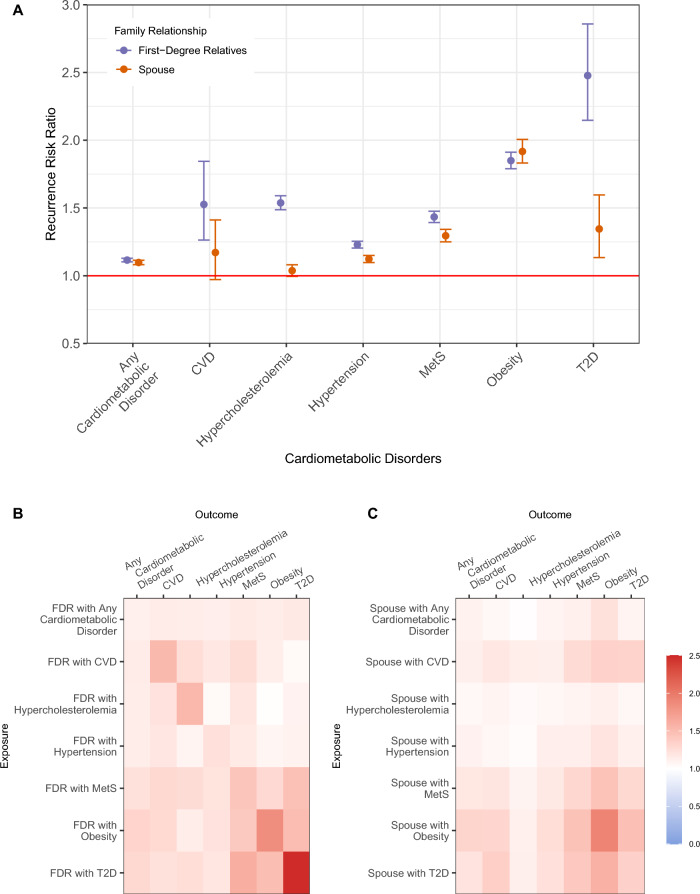
Familial aggregation (**A**) and co-aggregation of cardiometabolic disorders among first-degree relatives (**B**) and spouses (**C**), adjusted for age, age^2^, and sex. Intensity of the colours in figure B and C indicates the magnitude of recurrence risk ratio for cardiometabolic disorders indicated in the X-axis (outcome) by having family member with cardiometabolic disorders indicated in Y-axis (exposure). CVD: any cardiovascular disease defined by at least one of four types of cardiovascular diseases; FDR: first-degree relative; MetS: metabolic syndrome; T2D: type 2 diabetes

Positive familial aggregation of cardiometabolic disorders is shown in Fig. 2A; having an FDR or a spouse affected with a certain cardiometabolic disorder associated with a higher risk of the same disorder (ranging from λ_FDR_: 1.23 (95% CI 1.20–1.25) for hypertension, to λ_FDR_: 2.48 (95% CI 2.15–2.86) for T2D; and ranging from λ_spouse_: 1.04 (95% CI 1.00–1.08) for hypercholesterolemia, to λ_spouse_: 1.92 (95% CI 1.83–2.01) for obesity; details in Additional file 2: Table S2). In general, the recurrence risk ratios in individuals with an affected FDR were substantially higher than the recurrence risk ratios in individuals with an affected spouse (λ_FDR_ > λ_spouse_). The exception was obesity, for which there was a modestly lower recurrence risk ratio among FDR than spouses (λ_FDR_: 1.85 (95% CI 1.79–1.91) < λ_spouse_: 1.92 (95% CI 1.83–2.01)). In addition to these analyses, we also calculated the familial aggregation of extended CVD and found slightly lower familial aggregation of this extended CVD compared to the original CVD (λ_FDR_: 1.22 (95% CI 1.16–1.28) and λ_spouse_: 1.08 (95% CI 1.01–1.15) for extended CVD, λ_FDR_: 1.53 (95% CI 1.26–1.84) and λ_spouse_: 1.17 (95% CI 0.97–1.41) for the original CVD; details in Additional file 2: Table S3).

Additionally, we performed exploratory stratified familial aggregation analysis to examine possible differential effects of kinship type (affected sibling vs. affected offspring vs. affected parent), sex (men vs. women), and age (age < 40 vs. 40–60 vs. > 60 years). The group with ≥ 1 affected offspring showed the highest recurrence risks, followed by the group with ≥ 1 affected sibling. The group with ≥ 1 affected parent showed the lowest recurrence risks (details in Additional file 2: Table S6). Sex-stratified analyses did not show differences in recurrence risks between men and women (details in Additional file 2: Table S7). Age stratification showed generally similar recurrence risks across age categories. Only recurrence risk of T2D in the group age < 40 seemed higher than in older age categories, although this estimate is relatively imprecise and possibly inflated due to low T2D prevalence in this category (details in Additional file 2: Table S8).

### Familial co-aggregation

All pairs of cardiometabolic disorders showed positive familial co-aggregation among FDR (Fig. 2B), as well as spouses (Fig. 2C); either having FDR or a spouse affected with a certain cardiometabolic disorder conferred a higher risk of other cardiometabolic disorders. Significantly elevated recurrence risks of MetS, obesity, and T2D were observed among individuals with a first-degree relative affected by MetS, obesity, or T2D. Although the co-aggregation of the disorders was positive in both FDR and spouses, most of the co-aggregation of the disorders between FDR were higher than spouses (λ_FDR_ > λ_spouse_). In spouses, obesity showed a higher co-aggregation with other disorders than in FDR. This higher co-aggregation in spouses than in FDR was also observed in pairs of CVD-MetS and CVD-T2D. In addition, using the extended CVD definition resulted in positive familial co-aggregation, yet with a lower lambda compared to the original CVD definition (from λ_FDR_: 1.06 (95% CI 1.02–1.10) for hypercholesterolemia to λ_FDR_: 1.12 (95% CI 1.08–1.16) for MetS, and from λ_spouse_: 1.05 for hypertension to λ_spouse_: 1.16 for obesity and T2D; details in Additional file 2: Table S3). Unexpectedly, for extended CVD we observed no familial co-aggregation of risk of T2D (λ_FDR_: 1.00 (95% CI 0.92–1.08)).

We estimated λ_R_ of cardiometabolic disorders in FDR and spouses affected with one or more cardiometabolic disorders (i.e. ‘any cardiometabolic disorders’). We found a similar co-aggregation pattern, that is, those with a FDR with any cardiometabolic disorders had a higher recurrence risk (1.13 for both obesity and hypertension to 1.17 for T2D; see Additional file 2: Table S2), which were higher than for spouses (λ_spouse_ 1.08 for hypertension and T2D).

### Heritability

**Fig. 3:**
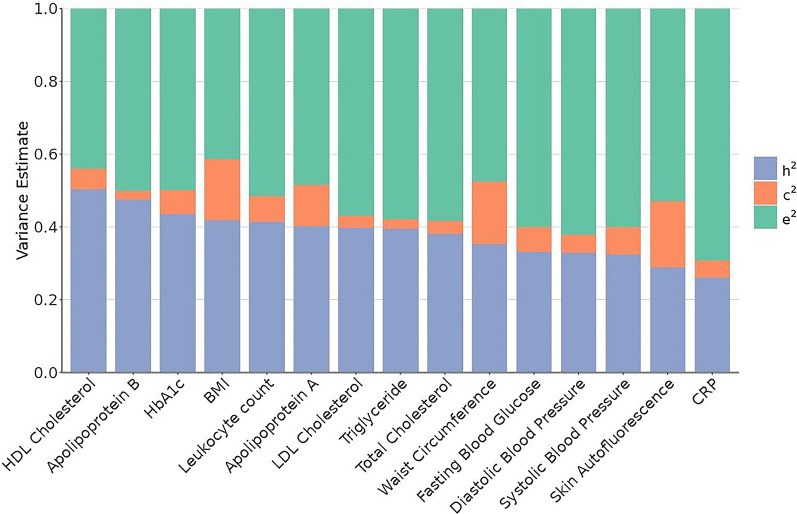
Variance component of cardiometabolic biomarkers, adjusted for age, age^2^, and sex. Y-axis indicates the estimates of variance components of the cardiometabolic traits indicated in the X-axis. h^2^: variance of cardiometabolic traits due to genetic (Va/Vp), also known as heritability, c^2^: variance of cardiometabolic traits due to shared (or common) environment (Vc/Vp), e^2^: variance of cardiometabolic traits due to unique environment (Ve/Vp)

Cardiometabolic traits had moderate levels of heritability (h^2^_CRP_: 0.26 to h^2^_HDL_: 0.50). Heritability explained much more variance than the shared environment in each cardiometabolic trait (c^2^_Apolipoprotein B_: 0.02 to c^2^_Skin autofluorescence_: 0.18; see Fig. 3, details in Additional file 2: Table S4). Together with a substantial heritable component, BMI and waist circumference had the largest estimates for the shared environmental variance component (i.e., c^2^: 0.17) compared to other cardiometabolic traits. Furthermore, we performed sensitivity analysis using traditionally log-transformed cardiometabolic traits, yielding similar results (Additional file 1: Fig. S3).

### Phenotypic and genetic correlation

**Fig. 4:**
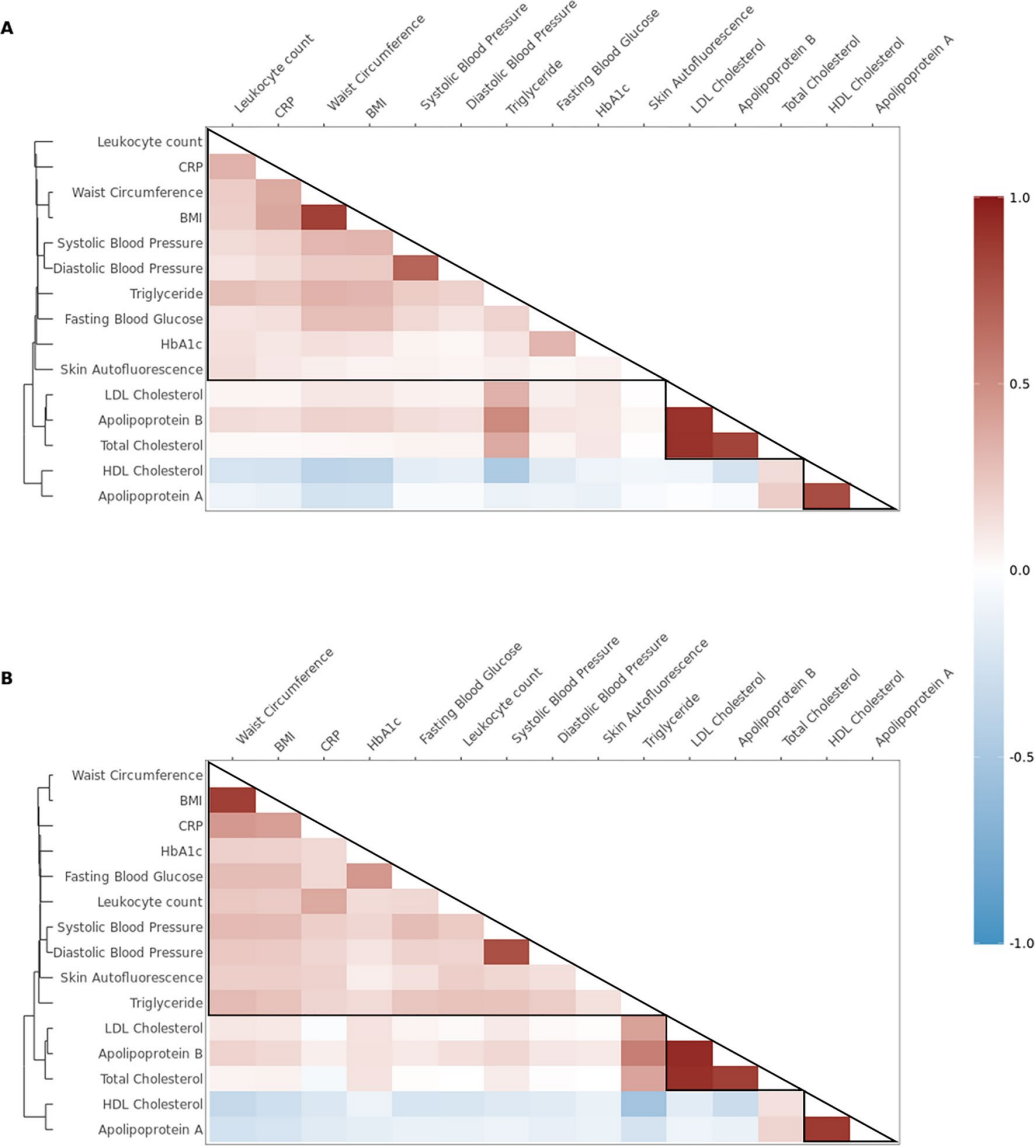
Phenotypic (**A**) and genetic (**B**) correlations between pairs of wide range of cardiometabolic traits. Dendrogram on the left side represents hierarchical distance between cardiometabolic traits. Clustering of cardiometabolic traits was performed using k-means clustering method, which generated three clusters indicated by the highlighted triangles. Phenotype and genetic correlations were adjusted for age, age^2^, sex, and shared common environment

Cardiometabolic traits showed a wide range of phenotypic (range from r_P HDL-Triglyceride_: − 0.47 to r_P Apolipoprotein B-LDL_ and r_P total cholesterol-LDL_: 0.91) and genetic correlations (range from r_G HDL-Triglyceride_: − 0.53 to r_G Apolipoprotein B-LDL_: 0.94), as shown in Fig. 4. Additional information can be found in Table S5 in Additional file 2. When we applied unsupervised hierarchical clustering, these correlations showed strong clustering of traits: (1) HDL cholesterol and apolipoprotein A, and (2) LDL cholesterol, apolipoprotein B, and total cholesterol. A third cluster included the remaining traits. Within this cluster, increased clustering was seen between obesity traits (i.e., BMI and waist circumference) and blood pressure traits (i.e., SBP and DBP). Although included in the third cluster together with other non-lipid traits, phenotypic correlations between skin autofluorescence and other traits were practically absent. In addition, weak-to-moderate genetic correlations were identified between triglycerides with glucose markers, blood pressure, and obesity markers (from r_G Triglyceride-HbA1c_: 0.16 to r_G Triglyceride -waist circumference_: 0.30). We also explored the environmental correlations across various cardiometabolic traits (Additional file 1: Fig. S4, details in Additional file 2: Table S5).

## Discussion

In this study, we aimed to estimate the genetic and environmental contribution to the co-occurrence of cardiometabolic disorders and traits within families, using objective measurements in a large multi-generational family study. We quantified the familial (co-)aggregation of six cardiometabolic disorders in first-degree relatives and spouses. Individuals with a first-degree relative affected with one of the cardiometabolic disorders had a higher risk of having the same or related disorders. Similarly, individuals with a spouse affected with cardiometabolic disorders had a higher risk of having the same or related disorders, suggesting an effect of shared environmental factors and/or assortative mating. Also, we estimated the heritability of fifteen cardiometabolic traits. These cardiometabolic traits had moderate heritability, indicating a role for genetics underlying the recurrence of cardiometabolic disorders within a family. Finally, we found moderate genetic correlations between cardiometabolic traits, suggesting genetics as an important but not exclusive underlying mechanism of the interrelation between cardiometabolic disorders.

Familial aggregation is evidence for a role of shared genetics and shared environment within a family in the occurrence of complex disorders. Positive familial aggregation of cardiometabolic disorders has been suggested by previous studies [10–17, 21]. Consistent with the literature, our study also found positive familial aggregation between first-degree relatives although of somewhat lower magnitude. For example, in The Framingham Offspring Study, risks of CVD was approximately 2 times higher in middle-aged adults with at least one parent with CVD [10] and 1.5 time if they had at least one sibling with CVD [11]. For T2D, a large Danish study also identified an up to 3.4 times higher risk in first-degree relatives than the general population [36], even higher than our estimate of 2.48 higher risk in first-degree relatives. Other studies also found higher familial aggregation of MetS compared to our recurrence risk estimate λ_FDR_ = 1.43 (95% CI 1.39–1.48) in individuals with affected first-degree relatives. A large population-based study in China identified a two to three times higher risk of MetS in younger siblings if their eldest sibling was affected by MetS [15]. Also, in the Tehran Lipid and Glucose Study, the risk of MetS was higher among offspring with affected parents (OR: 2.29–4.53) [16]. A possible explanation for the varying aggregation estimates is the heterogeneity between studies due to different family relationship included, age diversity between studies, and differences in ethnicity, lifestyle, and health behaviors between the Netherlands and other countries. Another possible explanation may arise from the diverse definitions of different disease phenotypes. In our study, we utilized both an extended CVD phenotype and a narrow CVD phenotype, the latter encompassing four CVD types: myocardial infarction, heart failure, stroke, and cardiac surgery. We observed a slightly lower familial aggregation in the extended CVD phenotype. This difference could be attributed to varying levels of heterogeneity in the extended CVD phenotype compared to the narrow CVD phenotype, with the latter demonstrating greater homogeneity. Despite these differences, the evidence converges on a major role of shared genetics in determining cardiometabolic risk. In exploratory analysis, we observed a higher recurrence risk in individuals with affected siblings and affected offspring when compared to individuals with affected parents, although possibly this is driven by age differences between offspring and parents. However age-stratified exploratory analysis showed that the recurrence risk estimates were stable across both age and sex.

We considered recurrence risk estimates in spouses a negative control to those in family: given that spouses are unlikely to be genetically related, estimates of spousal recurrence reflect the effects of shared environment and/or assortative mating on cardiometabolic risk. A Danish study observed around 1.5 times higher risk of T2D among individuals with a spouse affected with T2D [36], and in previous work in both Japanese and Dutch, we corroborated higher spousal cardiometabolic risk, for T2D (OR: 1.20 vs 1.59), hypertension (OR: 1.34 vs 1.45), and MetS (OR: 1.77 vs 1.77) in Japanese vs Dutch [37]. Although using the same cohort study, the estimates for spousal concordance in the Dutch Lifelines Population [37] are slightly higher than our current familial aggregation estimates, which may be attributed to differences in the statistical approaches used to estimate the risk of disorders among spouses. The spousal concordance was related to concordance in lifestyle factors, such as physical activity, smoking, and alcohol drinking, indicative of potential cohabitation effects, and assortative mating [37–39].

We found that cardiometabolic traits had moderate genetic extent in which the heritability estimates are consistent with those in the literature although some variation can occur due to differences in age, ethnicity, study design [40], and type of measurement used [41]. For example, slightly different estimates of heritability were found in a family study in a 1564 Chinese individuals from 494 families [20], reporting heritability of fasting glucose (h^2^: 0.17), waist circumference (h^2^: 0.26), SBP (h^2^: 0.24), DBP (h^2^: 0.17), triglycerides (h^2^: 0.41), HDL-C (h^2^: 0.49), LDL-C (h^2^: 0.47), total cholesterol (h^2^: 0.46), CRP (h^2^: 0.38), and BMI (h^2^ = 0.38). Also, a Dutch twin study presented moderate to high heritability for a range of cardiometabolic traits from 0.47 for insulin level to 0.78 for BMI [42], with estimates typically being higher than family studies including the present study.

Genetic similarity between first-degree relatives is likely to contribute to familial co-aggregation of related cardiometabolic disorders [21–24]. A previous study found that parental history of one or more CVD (i.e., myocardial infarction, stroke, and angina) at a younger age < 55 year in the father and < 65 year in the mother significantly increased the risk of MetS in women, with ORs from 1.62 to 1.84 [21]. Another family study identified increased risk of having multiple cardiometabolic disorders in relation to parental history of diabetes (OR: 1.54, 95% CI 1.01–2.33) and parental history of hypertension (OR 1.42, 95% CI 1.06–1.91). The risk was even higher when both parents were affected with hypertension or diabetes, suggesting an additive genetic effect on the risk of cardiometabolic disease co-occurrence [24]. Similarly, a population-based study in US found a higher risk of co-occurring cardiometabolic disorders when individuals had a family history of diabetes or hypertension, and only a slightly increased risk with family history of obesity [23]. These studies mostly found evidence for co-aggregation between CVD, T2D, and hypertension. Compared to these studies, our estimates of co-aggregation between obesity, T2D, and MetS were larger, while hypertension, CVD, and hypercholesterolemia showed only modest familial co-aggregation. A possible reason for this difference is that previous studies used mostly self-reported family history without actual validation with objective laboratory measures in family members.

Cardiometabolic disorders likely share pathophysiological mechanisms such as inflammation and insulin resistance [4–6]. The high insulin resistance in obesity and diabetes is thought to induce inflammation, causing vascular damage and endothelial dysfunction. Such vascular damage and dysfunction lead to increased production of vasoconstrictors, and subsequently to an increase in vascular resistance, a major contributor to CVD and hypertension [5, 6]. Consistent with this, we found significant phenotypic and genetic correlations between blood pressure, obesity traits, inflammatory markers, and fasting glucose, although these correlations were of modest strength. In the present study, we also found modest genetic correlations between HDL cholesterol and triglyceride with glucose markers, blood pressure, and obesity, which are the traits used for the definition of MetS. The accumulation of Advanced Glycation End products has been considered a potential cross-link between diabetes and cardiovascular events, by increasing inflammation and causing endothelial dysfunction [43, 44]. We found little evidence of this in the present study: we observed only weak correlations between skin autofluorescence and other cardiometabolic traits, although their genetic correlations are slightly higher than the phenotypic correlation. Furthermore, the association of skin autofluorescence, CVD, and T2D in the previous studies in this population were independent of glucose markers [43, 45].

Strengths of this study are that it is the largest family and comprehensive study of cardiometabolic outcomes to date, investigating six interrelated cardiometabolic disorders and fifteen intermediate cardiometabolic traits. It was conducted in a large-scale population-based, multi-generational cohort, representative of the general Dutch population [46]. We used a combination of objective laboratory measurements, questionnaire data, and medication data, resulting in precise outcome definitions and thus precise estimates of recurrence risk and heritability. Our study provides insights into the genetic and/or environmental mechanisms that underly of co-existing cardiometabolic disorders within individuals and families. Our findings highlight the role of shared genetics and environmental factors on the risk of cardiometabolic disorders and suggests overlapping genetic structure between disorders. Furthermore, our estimates of recurrence risk may inform clinicians and health services in diagnosis, patient communication, and potential screening efforts based on family history. However, several study limitations need to be addressed as well. Firstly, only data were available on family members participating in Lifelines. Missing data on non-participating family members may have caused underestimation of recurrence risk and heritability. Secondly, although largely representative of the Dutch population Lifelines predominantly consists of Dutch participants; generalization to other ancestries and maybe even to other European populations is therefore uncertain.

## Conclusion

To conclude, in our large multi-generational family study, cardiometabolic disorders show positive (co-)aggregation within families and to a lesser extent between spouses. We found moderate heritability for a wide variety of intermediate cardiometabolic traits and moderate genetic correlations between traits. These results suggest that genetic factors are an important but moderate contributor to the co-occurrence of cardiometabolic traits. We find evidence for a strong contribution of shared environmental factors, especially for obesity. To further elucidate potential mechanisms for co-aggregation, future studies may focus on identifying the shared genetic factors, the specific shared environmental risk factors, and potential gene-environment interaction.

### Supplementary Information

**Additional file 1:** Supplementary Methods. **Figure S1**. Morbidities in cardiometabolic disorders. **Figure S2** Prevalence of cardiometabolic disorders per age category. **Figure S3** Heritability estimates of log-transformed cardiometabolic traits. **Figure S4** Correlations between cardiometabolic traits due to shared environment.

**Additional file 2: Table S1**. Overlapping cardiometabolic disorders in adults participants (N=152,723). **Table S2**. Familial aggregation and coaggregation of cardiometabolic disorders (N=152,723). **Table S3**. Familial aggregation and coaggregation of cardiometabolic disorders using extended definition of cardiovascular diseases (N=152,723). **Table S4**. Variance component estimates (N=162,416). **Table S5**. Phenotypic, genetic and environmental correlation of cardiometabolic traits (N=162,416) **Table S6**. Familial aggregation of cardiometabolic disorders in first-degree relatives stratified by familial kinship (N=152,723). **Table S7**. Familial aggregation of cardiometabolic disorders in first-degree relatives stratified by sex (N=152,723). **Table S8**. Familial aggregation of cardiometabolic disorders in first-degree relatives stratified by age (N=152,723). **Table S9**. Explanation of different ATC codes used in the study.

## Data Availability

Lifelines data may be obtained from a third party and are not publicly available. Researchers can apply to use the Lifelines data used in this study. More information about how to request Lifelines data and the conditions of use can be found on their website (https://www.lifelines.nl/researcher/how-to-apply).

## Abbreviations

BMI: Body mass index
CVD: Cardiovascular diseases
CRP: C-reactive protein
c^2^: Contribution of household
DBP: Diastolic blood pressure
FDR: First-degree relatives
HbA1c: Glycated haemoglobin
HDL: High density lipoprotein
h^2^: Heritability
LDL: Low density lipoprotein
MetS: Metabolic syndrome
REML: Residual maximum likelihood
r_G_: Genetic correlation
r_P_: Phenotypic correlation
SBP: Systolic blood pressure
T2D: Type 2 diabetes
λ_R_: Recurrence risk ratios
