# Supplementary material for: Familial co-aggregation and shared genetics of cardiometabolic disorders and traits: data from the multi-generational Lifelines Cohort Study
Source: Cardiovasc Diabetol. 2023 Oct 21;22:282. doi: 10.1186/s12933-023-02017-w (PMC10590015; doi:10.1186/s12933-023-02017-w)
Supplement: Supplementary file 1 — Additional file 1: Supplementary Methods. Figure S1. Morbidities in cardiometabolic disorders. Figure S2 Prevalence of cardiometabolic disorders per age category. Figure S3 Heritability estimates of log-transformed cardiometabolic traits. Figure S4 Correlations between cardiometabolic traits due to shared environment. [file 12933_2023_2017_MOESM1_ESM.docx]

### **Familial Co-aggregation and Shared Genetics of Cardiometabolic Disorders and Traits: Data from The Multi-Generational Lifelines Cohort Study**

### Rima D. Triatin^†^, Zekai Chen^†^, Alireza Ani, Rujia Wang, Catharina A. Hartman, Ilja M. Nolte, Chris H.L. Thio*, Harold Snieder*

### **Electronic Supplementary Material**

**Additional file 1**

**Content:**

**Supplementary Methods**

**Supplementary Figures**

**Figure S1.** Morbidities in cardiometabolic disorders.

**Figure S2** Prevalence of cardiometabolic disorders per age category.

**Figure S3** Heritability estimates of log-transformed cardiometabolic traits.

**Figure S4** Correlations between cardiometabolic traits due to shared environment.

### **Supplementary Methods**

**Study Population**

The recruitment of the Lifelines study was family-based by design. Eligible subjects between the ages of 20 and 50 were invited to participate through their general practitioner. After the inclusion of these individuals, their partner, children, parents and partner’s parents were also invited to participate in the study. Additionally, single individuals could register for participation online. In this way a three-generation family study was realized. We used the information on family members as well as information on (anonymized) names and birth dates of parents provided by all participants in questionnaires to define family relationships in Lifelines and verified where possible in participants with genetic data (n~80,000). In total, there were 30,914 families (of size ≥2) of up to four generations and 40,496 singletons (i.e., participants without any relative participating in Lifelines). The largest family connected 189 participants. Spouses without children were considered as a family of size 2, even though they are genetically unrelated. Participants who live in the same house shared the same household ID. Household was defined based on the self-reported questionnaire, family pedigree, and their postcode. The question is “Who are living in your house (more than half the time)? My partner/ father/ mother/ children/ brothers/ sisters/ I live alone”. For participants who lived together with family members, and their family members participated in the Lifelines, and they had the same postcode, we assigned them the same household ID. Otherwise, we assigned them a unique household ID. For participants who reported they live alone, we also assigned them a unique household ID.

### **Continuous Cardiometabolic traits**

Fasting blood was collected from all participants aged 8 years and older at the baseline visit. Of these blood samples, glucose markers (i.e., fasting blood glucose and HbA1c) and lipid markers, consisting of total cholesterol, HDL-cholesterol, LDL-cholesterol, triglycerides, apolipoprotein A1, and apolipoprotein B100, were measured using the Roche Modular P chemistry analyser. Also, leukocytes were counted from blood samples collected in EDTA tubes using the Sysmex XE2100.

High-sensitivity CRP (hsCRP) levels were determined in blood samples using three different methods: (1) hsCRP assessed in serum using CardioPhase hsCRP (Siemens Healthcare Diagnostics, Marburg, Germany); (2) hsCRP assessed in plasma using CardioPhase hsCRP (Siemens Healthcare Diagnostics, Marburg, Germany); (3) hsCRP assessed in plasma using CRPL3 (Roche Diagnostics, Mannheim, Germany). Method 2 and 3 are identical and only differ in terms of the manufacturer. To make method 1 comparable with methods 2 and 3, a conversion formula (0.92 x value - 0.01) was applied to the values of method 1 [1]. All blood assessments were performed at the laboratory centre of the University Medical Center Groningen.

Besides, skin autofluorescence was measured in adults during baseline visits to quantify the accumulation of Advanced Glycation End products (AGE) in the skin. Using the AGE Reader (Diagnoptics Technologies, Groningen, the Netherlands), measurements were made by illuminating a skin surface at the volar side of the forearm, 10 cm below the elbow, at room temperature, for 10 seconds. Three measurements were made and the average of these three values (or the median in case of 1 deviant value) is used for analyses [2].

Physical measurements were performed by a trained research nurse, following the Lifelines protocol [3]. Body weight, body height, and waist circumference were measured in standing position. Meanwhile, SBP and DBP were measured repetitively 10 times within 10 minutes while seated with an automatic blood pressure monitor (DinaMap, PRO 100V2) using the appropriate cuff size according to the arm circumference of the participant. The average of the last 3 measurements was used for analysis.

### **Heritability, Genetic Correlation and Phenotypic Correlation**

To test the significance of the genetic correlations (|r_G_|>0) and phenotypic correlations (|r_P_|>0), the model in which all correlations were estimated was compared, using a likelihood-ratio test, to a model in which genetic correlations and phenotypic correlations for all measures were constrained to be zero. Similarly, genetic correlations and phenotypic correlations were constrained to be equal to 1 or −1 to test the presence of complete overlap of genetic effects (|r_G_|=1) and phenotypic effects (|r_P_|=1).

**Supplementary Figures**


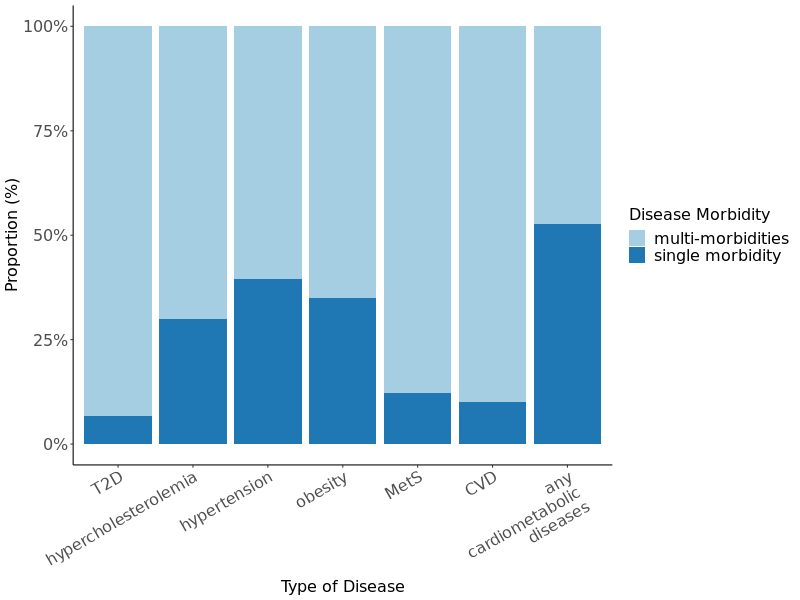


**Figure S1.** Morbidities in cardiometabolic disorders (N=66,695). Data are presented as proportion (%) of disease morbidity over total cases of each disease.


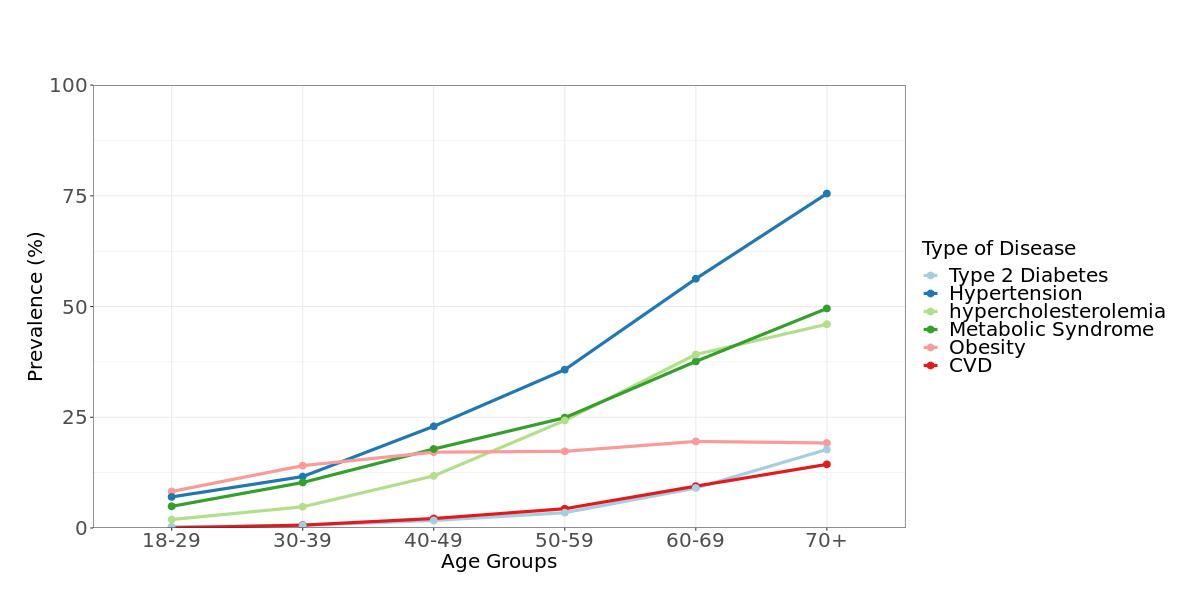


**Figure S2** Prevalence of cardiometabolic disorders per age category. CVD, cardiovascular diseases.


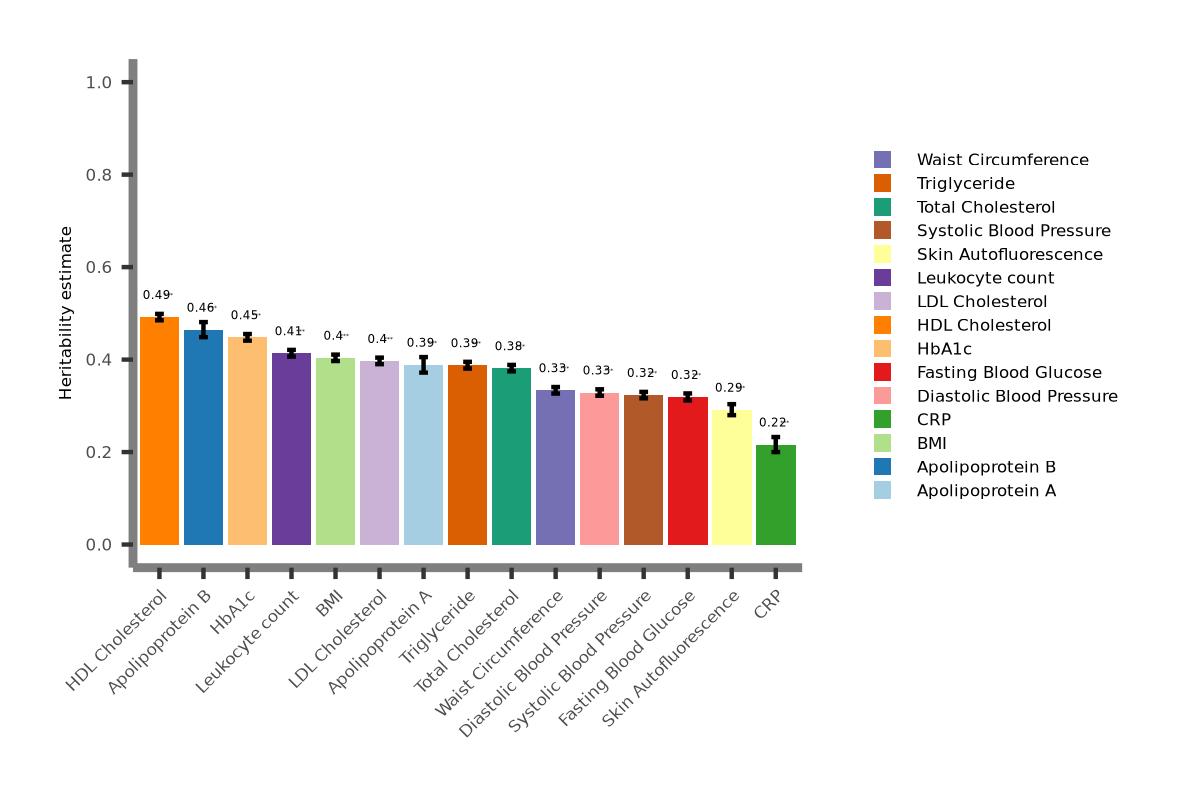


**Figure S3** Heritability estimates of log-transformed cardiometabolic traits. Estimates were adjusted for age, age^2^, sex and shared common environment. Error bar and significance level represent the standard error and significance level of heritability estimates over h^2^=0, respectively. BMI, body mass index; CRP, c-reactive protein; HbA1c, glycated haemoglobin; HDL, high-density lipoprotein; LDL, low-density lipoprotein. *p-value<0.05, **p-value<0.01, ***p-value<0.001.


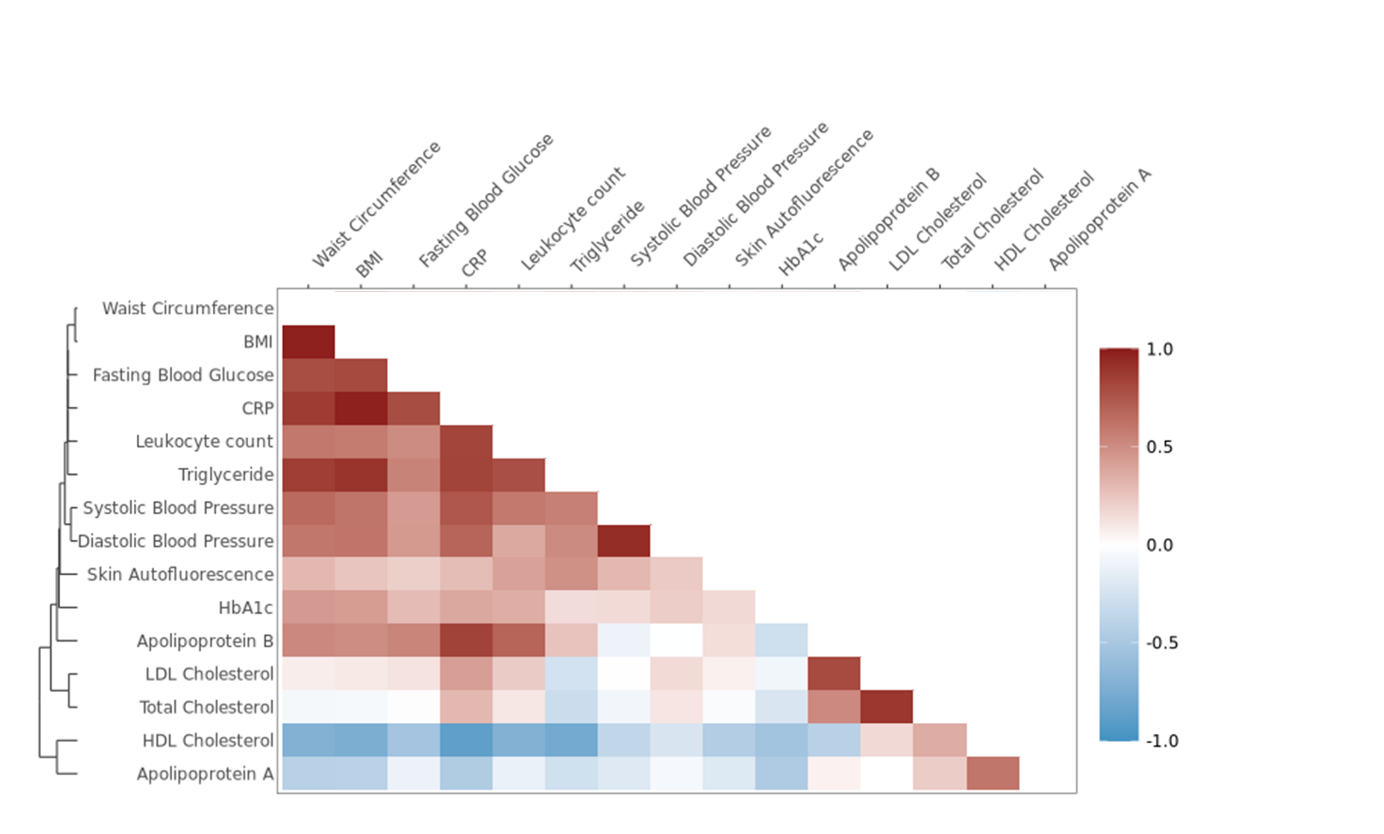


**Figure S4** Correlations between cardiometabolic traits due to shared environment. Correlation coefficients are adjusted for age, age^2^, sex, and family pedigree. Dendogram on the left side showed the hierarchical clustering between cardiometabolic traits due to shared environment. BMI, body mass index; CRP, c-reactive protein; HbA1c, glycated haemoglobin; HDL, high-density lipoprotein; LDL, low-density lipoprotein.

**Reference**

1. Mac Giollabhui N, Alloy LB, Schweren LJS, Hartman CA: Investigating whether a combination of higher CRP and depression is differentially associated with worse executive functioning in a cohort of 43,896 adults. Brain Behav Immun 2021, 96:127-134.

2. van Waateringe RP, Slagter SN, van der Klauw MM, van Vliet-Ostaptchouk JV, Graaff R, Paterson AD, Lutgers HL, Wolffenbuttel BH: Lifestyle and clinical determinants of skin autofluorescence in a population-based cohort study. Eur J Clin Invest 2016, 46(5):481-490.

3. Scholtens S, Smidt N, Swertz MA, Bakker SJ, Dotinga A, Vonk JM, van Dijk F, van Zon SK, Wijmenga C, Wolffenbuttel BH et al: Cohort Profile: LifeLines, a three-generation cohort study and biobank. Int J Epidemiol 2015, 44(4):1172-1180.
